# Protective effects of inhaled antioxidants against air pollution-induced pathological responses

**DOI:** 10.1186/s12931-023-02490-7

**Published:** 2023-07-13

**Authors:** Kevin D. Schichlein, Gregory J. Smith, Ilona Jaspers

**Affiliations:** 1 Curriculum in Toxicology and Environmental Medicine, University of North Carolina at Chapel Hill, 116 Manning Drive, Chapel Hill, NC 27599-7310 USA; 2 Department of Genetics, University of North Carolina at Chapel Hill, Chapel Hill, NC 27599 USA; 3 Center for Environmental Medicine, Asthma, and Lung Biology, University of North Carolina at Chapel Hill, Chapel Hill, NC 27599 USA; 4 Department of Pediatrics, University of North Carolina at Chapel Hill, Chapel Hill, NC 27599 USA

**Keywords:** Air pollution, Antioxidant, Inhalation, Airway, Intranasal, Intrapulmonary, Dietary, Vitamins

## Abstract

As the public health burden of air pollution continues to increase, new strategies to mitigate harmful health effects are needed. Dietary antioxidants have previously been explored to protect against air pollution-induced lung injury producing inconclusive results. Inhaled (pulmonary or nasal) administration of antioxidants presents a more promising approach as it could directly increase antioxidant levels in the airway surface liquid (ASL), providing protection against oxidative damage from air pollution. Several antioxidants have been shown to exhibit antioxidant, anti-inflammatory, and anti-microbial properties in in vitro and in vivo models of air pollution exposure; however, little work has been done to translate these basic research findings into practice. This narrative review summarizes these findings and data from human studies using inhaled antioxidants in response to air pollution, which have produced positive results, indicating further investigation is warranted. In addition to human studies, cell and murine studies should be conducted using more relevant models of exposure such as air–liquid interface (ALI) cultures of primary cells and non-aqueous apical delivery of antioxidants and pollutants. Inhalation of antioxidants shows promise as a protective intervention to prevent air pollution-induced lung injury and exacerbation of existing lung disease.

**Graphical Abstract:**

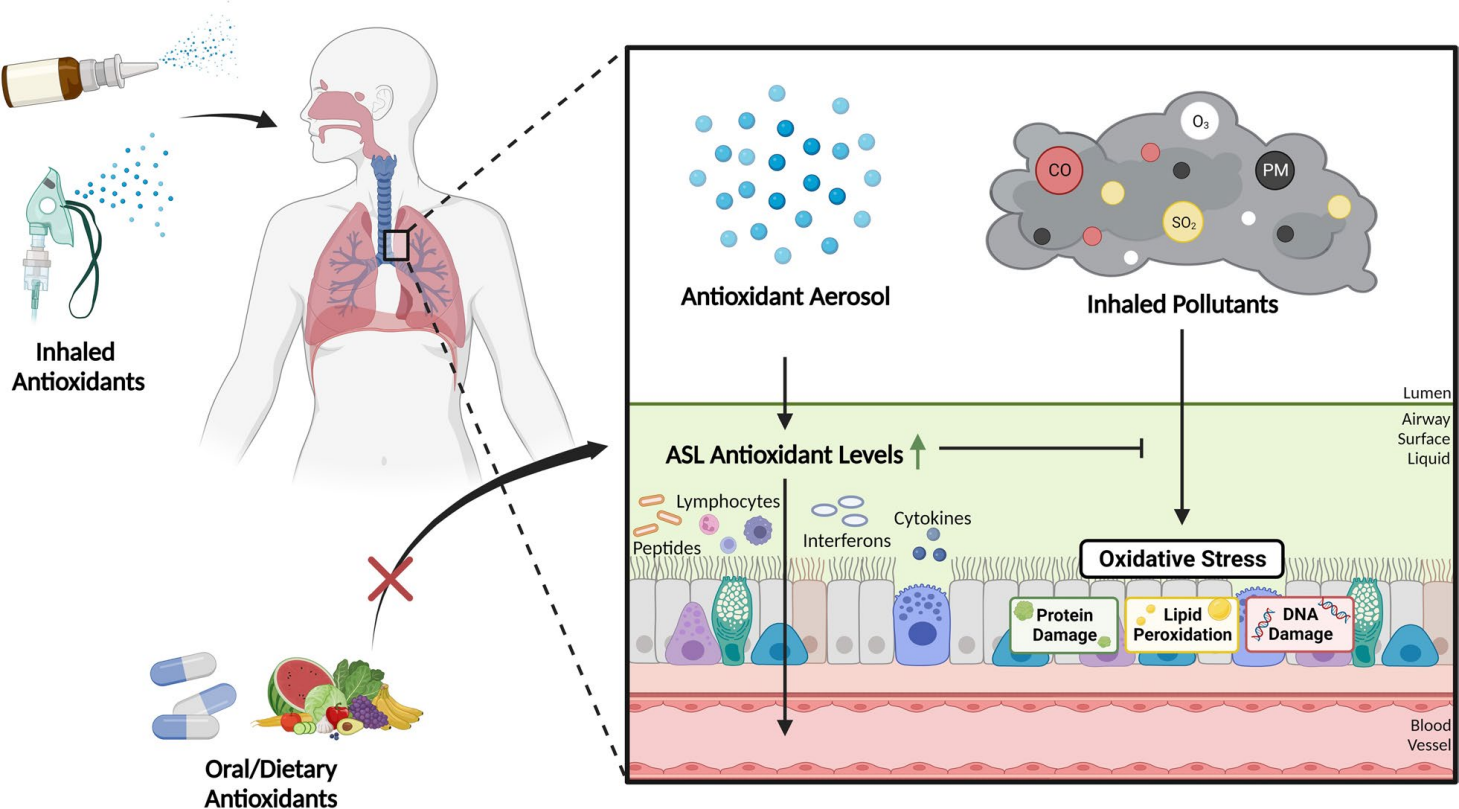

## Introduction

Long-term exposure to air pollution is associated with increased risk of cardiopulmonary and neurological diseases, cancer, and overall mortality [1]. While long-term exposure increases the risk of lung diseases such as chronic obstructive pulmonary disease (COPD) and asthma, especially in adolescents, short-term exposure can cause airway inflammation, hyperreactivity, decreased pulmonary function, susceptibility to microbial infection, and exacerbation of existing lung diseases. In 2019 the World Health Organization (WHO) found that 99% of the world population live in places where WHO air quality guidelines are not met, and caused an estimated 4.2 million premature deaths worldwide resulted from ambient air pollution, with low-and middle-income countries accounting for 89% of the estimated deaths [2]. Given the ever-increasing burden of air pollution, new strategies to mitigate its adverse health effects are needed. Further investigation into dietary and pharmacologic interventions to mitigate air pollution-induced adverse health effects may provide strategies for public health officials to overcome these challenges. One promising strategy is to increase the concentration of antioxidants (e.g., vitamin E, glutathione, etc.) in the lung, particularly at the surface of the respiratory epithelium, to counteract air pollution-induced oxidative stress. This narrative review will discuss the biochemical basis of this strategy, current research, and mixed results of studies using oral and dietary delivery of antioxidants. As future direction, we present evidence showing that treatment using several antioxidants through respiratory delivery, both intranasal and intrapulmonary, may offer improved success over oral and dietary delivery.

### Composition of air pollutants and mechanisms of lung injury

Air pollution consists of gaseous components (ozone, volatile organic compounds, carbon monoxide, nitrogen oxides) and particulate matter (PM). PM is classified by particle size, ranging from ultrafine (PM_0.1_), fine (PM_2.5_), to coarse (PM_10-2.5_). PM_0.1_, particles with an aerodynamic equivalent diameter (AED) ≤ 0.1 µm, have high surface area, travel deep into the small airways, and can reach systemic circulation, making them especially harmful to inhale. In general, smaller particles (< 10 µm) can reach the lower airways and larger particles (> 10um) mostly deposit in the upper airways [3]. PM can be composed of metals, carbon, sulfates, nitrates, polycyclic aromatic hydrocarbons (PAHs), biological compounds, and can also form secondary PM through the nucleation and coagulation of gaseous pollutants onto primary PM [4]. Sources of primary PM include natural processes such as volcanic eruptions, wildfires, erosion, and through anthropogenic processes such as cigarette smoke, traffic, mining, construction, farming, power plants, and any process involving combustion of fuel [4].

Air pollution composition is heterogenous and varies across regions and with atmospheric aging; however, one of the unifying characteristics of inhaled PM and gases is their potential to cause oxidative stress in the airway epithelium [5]. Oxidative stress occurs when oxidation–reduction homeostasis is perturbed by an accumulation of reactive oxygen species (ROS) and reactive nitrogen species (RNS) [6]. ROS and RNS are endogenously produced by inflammatory cells, during cellular respiration, and by enzymes, but are carefully controlled by antioxidant systems. Introduction of oxidants, free radicals, or redox catalysts through inhalation of air pollutants can overwhelm these systems, resulting in damage to DNA, membranes, and proteins via oxidation and eventually cytotoxicity [6]. Transition metals found in particulate matter are also capable of generating further oxidants through Fenton-like reactions [7].

### Interactions of air pollutants with the respiratory mucosa

The respiratory system is a primary route of exposure to airborne environmental insults; as such, the respiratory mucosa has several mechanisms to protect against injury from inhaled toxicants. The initial line of protection is the airway surface liquid (ASL) layer, which acts as a physical barrier, helps expel pathogens through mucociliary clearance, and contains biochemical defenses [8]. ASL has two distinct physical layers, the superficial mucus layer, and the lower periciliary layer. The mucus layer traps inhaled pathogens with secreted mucins (MUC5AC, MUC5B) while the periciliary layer facilitates ciliary movement with the help of tethered mucins (MUC1, MUC4, MUC16) [9]. ASL also contains cytokines, antimicrobial peptides, antiviral interferons, leukocytes, and several types of antioxidants [10, 11].

High concentrations of antioxidants including antioxidant enzymes (dismutase, catalase, peroxidase, oxygenase) and small-molecule compounds (vitamin C, vitamin E, glutathione, uric acid, β-carotene) which act as free radical scavengers are present in the ASL [12]. Inhaled pollutants have been found to deplete ASL antioxidants and inhibit antioxidant enzymes, allowing for production of secondary oxidants through reaction with proteins, lipids, and carbohydrates in the ASL [13]. Because of this, it has been hypothesized that supplementation of ASL antioxidants through diet or inhalation could bolster antioxidant defenses and mitigate air-pollution induced oxidative stress.

### Search strategy

The literature search was performed using Pubmed/MEDLINE and Google Scholar with no time frame restriction using the following antioxidant search terms: antioxidant, vitamin C, ascorbic acid, vitamin E, tocopherol, vitamin D, calciferol, glutathione, GSH, N-acetyl cysteine, NAC; respiratory system search terms: lung, airway, nasal, inhalation, bronchial; and pollutant search terms: pollutant, diesel exhaust particle, DEP, house dust mite, HDM, wood smoke, smoke, ozone, gas, particulate matter, PM. These terms were used in the following combinations: [antioxidant term] AND [respiratory system term] AND [pollutant term], and if few results were found including air pollutant search terms, [antioxidant term] AND [respiratory system term].

### Scope of review

Given the topic of this review and the scarcity of literature on inhaled delivery of antioxidants, we chose to present this information as a narrative review. Introductory sections on air pollution and the respiratory mucosa are intended to provide succinct overviews of air pollution-induced pathological responses and justify the need for further investigation into inhaled supplementation of antioxidants. The discussion of human studies on oral supplementation was kept brief and largely summarized findings from previous reviews of the topic as the focus is on inhaled delivery. For antioxidants with few studies on protective effects against pollutants (vitamins C and E), studies of their effects on respiratory diseases, immune responses, and airway biology were included if links to air pollution exposure could be made.

### Vitamin D

Vitamin D, or cholecalciferol, is a nutrient involved in calcium regulation and phosphate homeostasis, playing a role in immune and musculoskeletal health. Vitamin D is obtained through diet and cutaneous synthesis from ultraviolet B radiation, followed by metabolism to its active form, 1,25-dihydroxyvitamin D, or calcitriol. Both cholecalciferol and calcitriol will be referred to as vitamin D in this review as primary airway cells are able to metabolize cholecalciferol into the active form [14]. Studies in cell lines often use calcitriol, as cell lines such as A549 cells express low levels of 1α-hydroxylase (CYP27B1), the enzyme responsible for vitamin D activation, and are unable to convert cholecalciferol to its active form [14].

Several studies have explored co- or pre-treatment with vitamin D in response to various pollutants. In cigarette smoke (CS)-exposed 16HBE cells, vitamin D exhibited anti-inflammatory effects, but not in primary hBECs [15]. 16HBEs treated with lipopolysaccharide (LPS) had attenuated ROS and DNA damage when co-treated with vitamin D, a result confirmed in a murine model. Serré et al. found that in LPS-exposed mice with normal vitamin D levels, nebulized treatment with vitamin D reduced inflammatory cells in bronchoalveolar lavage fluid (BALF) and significantly protected against epithelial barrier damage [16]. This study also found that vitamin D inhalation did not change serum vitamin D levels, indicating that inhalation could provide local therapeutic effects and minimal system effects. This is one of the few studies to investigate aerosolization of vitamin D to protect against exposure-induced inflammation.

The protective effects of vitamin D are most apparent in PM exposure. Transcriptome analysis of primary hBECs exposed to urban PM saw reduced expression of *IL6*, a gene encoding a pro-inflammatory cytokine, and increased expression of *G6PD*, an antioxidant pathway gene, with vitamin D treatment [17]. The same study also observed reduced lipid oxidation, increased antioxidant response, and reduced levels of IL-6. An earlier study from the same group found that urban PM caused an increase in pro-inflammatory T helper 17 (TH17) cell response driven by IL-23 in a myeloid dendritic cell-memory CD4^+^ T cell co-culture system. Co-exposure with vitamin D was able to attenuate this effect through a reduction in IL-17a^+^ and IFN-γ^+^ cells [18]. Tao et al. investigated lung injury in an in vivo (intramuscular dosing of mice) and in vitro (BEAS-2B) model through exposure to silicon dioxide-containing PM [19]. Both models showed induction of autophagy via Nrf2 after vitamin D treatment. Similar results were produced in another study using undifferentiated hBECs exposed to PM, showing reduced inflammation through the p38/NF-κB/NLRP3 pathway [20]. Reduced inflammation was also demonstrated by Bolcas et al. using a murine model of co-exposure to diesel exhaust particles (DEP) and HDM [21]. In this model, treatment with vitamin D attenuated accumulation of TH17 and TH2 cells and development of airway hyperresponsiveness. Vitamin D has been found to have therapeutic effects in models of epithelial barrier dysfunction, characteristic of COPD, caused by cigarette smoke and toluene diisocyanate [22, 23]. Vitamin D also suppressed development of pulmonary emphysema, epithelial‑mesenchymal transition (EMT), and fibrogenesis, all of which are associated with the development of COPD and are exacerbated by toxicant exposure [24, 25]. Altogether, these data indicate vitamin D has possible utility in preventing air pollution-induced oxidative stress, immune responses, and microbial infection.

Of the antioxidants discussed in the review, vitamin D is the most well-studied in the airways due to its antibacterial and antiviral properties. Vitamin D has been found to significantly increase gene expression and protein levels of cathelicidin, an antimicrobial peptide, in airway cell lines, immune cells, and primary airway epithelial cells (bronchial and tracheobronchial) [26–32]. Vitamin D provided protection from infection to rhinovirus, respiratory syncytial virus (RSV), influenza, and mycobacterium tuberculosis through suppression of inflammation and altered expression of viral and bacterial receptors. However, there are conflicting results on the effect of vitamin D on viral replication, a more functional marker of antiviral properties, between undifferentiated vs. fully differentiated human bronchial epithelial cells (hBECs) through media dosing [33].

In addition to the study from Serré et al., two other studies have investigated inhaled vitamin D. The studies did not involve pollutant exposure and instead looked at the ability of vitamin D to enhance neonatal lung maturation and to promote anti-tumor immune activity, where it was also found that despite exhibiting therapeutic effects in the lung, treatment did not affect serum levels of vitamin D or calcium, indicating inhaled vitamin D most likely does not pass through the epithelial membrane or cause hypercalcemia [16, 34, 35]. Mathyssen et al. expanded on this by examining the transcriptional profile of vitamin D associated enzymes in lung tissue [36]. They found that CYP24A1, the inactivating enzyme of vitamin D, was highly expressed in lung endothelial cells, preventing circulating vitamin D from reaching the lungs. Conversely, this could also explain why inhaled vitamin D does not reach circulation. In the same study it was shown that the vitamin D receptor was expressed in apical epithelial cells, making it an ideal target for inhaled delivery.

It is important to note that the status of vitamin D as an antioxidant is controversial and could not be confirmed in a recent systematic review [37]. It is possible that the protective effects of vitamin D are not produced through scavenging of free radicals or induction of antioxidant enzymes and are instead due to interactions with the vitamin D receptor. However, the studies presented here indicate that vitamin D has antioxidant properties in the lung.

### Glutathione

Glutathione (GSH) is a thiol tripeptide found at high concentrations in most cells and is the primary non-enzymatic antioxidant found in ASL [38]. The ratio of GSH, the reduced form of glutathione, to GSSG, the oxidized form, is a biomarker of cellular redox status, with the ratio dropping after exposure to oxidant stress [12, 39]. Notably, GSH is also the only antioxidant with a higher concentration in ASL than in plasma and is altered in several lung disease including cystic fibrosis (CF), idiopathic pulmonary fibrosis (IPF), and COPD [40]. Because of this, GSH inhalation has been evaluated as a treatment for these conditions.

A literature review looking specifically at inhaled GSH determined that it has potential as a treatment for cystic fibrosis (CF), chronic otitis media with effusion (OME), HIV seropositive individuals, IPF, and chronic rhinitis [40]. Randomized placebo-controlled trials found GSH improved oxygenation and clinical parameters in CF, although the effect may not be due to correction of oxidant/antioxidant balance. However, improvements in oxidant/antioxidant balance were observed in a nonrandomized IPF trial. Based on current evidence, inhaled GSH cannot be recommended for emphysema/COPD or asthma, the latter of which exhibited notable side effects (e.g., breathlessness, bronchoconstriction, cough), most likely due to co-occurrence of asthma and sulfite sensitivity. The author also recommended further research be conducted on other conditions linked to impaired antioxidant systems such as Farmer’s lung, multiple chemical sensitivity disorder, and exercise-induced oxidative stress. Although not mentioned, air pollution-induced lung injury, which occurs due to depletion of ASL antioxidants, could also potentially be inhibited by glutathione inhalation.

Several studies have explored the effects of polymorphisms in the genes encoding glutathione S-transferase (GST), an enzyme responsible for conjugation of GSH to xenobiotics, on air pollution exposure [41]. *GSTM1* and *GSTP1* polymorphisms have been associated with increased respiratory issues in response to ambient ozone and combined ragweed pollen/DEP exposure. The *GSTM1* null-phenotype was also shown to regulate DEP-induced inflammation in vitro [42]. Jaspers et al. found that GSH-ethylester was able to reverse DEP-induced susceptibility to influenza infection in well-differentiated respiratory epithelial cells [43].

Given that GSH concentrations initially decrease and then recover in ASL after air pollution exposure, it can be concluded that GSH is vital for first line defense against inhaled oxidants [13]. Despite this, GSH treatment has been sparingly investigated for mitigating the effects of air pollution exposure. Glutamine, a glutathione precursor, supplementation has been explored as a therapeutic for several lung diseases, most notably acute respiratory distress syndrome (ARDS); and despite promising results through parenteral administration, few studies have explored inhaled supplementation [44].

### N-acetylcysteine

N-acetylcysteine (NAC), another precursor to glutathione, is a prescription drug used to treat acetaminophen overdoses and as a mucolytic for muco-obstructive lung diseases [45]. NAC has direct antioxidant activity but also increases intracellular levels of cysteine which facilitates GSH synthesis [45]. NAC’s mucolytic properties stem from its the ability to break down disulfide bonds crosslinking mucus glycoproteins, reducing mucus viscosity and elasticity. This makes it an attractive option for inhaled treatment as it could potentially restore antioxidant capacity and alleviate mucus hypersecretion. Due to the number of benefits over glutathione, NAC has been investigated in several lung diseases and to protect against air pollution exposure.

Although taken orally for COPD, inhaled NAC has been approved for use in CF and has been shown to be effective as an adjunct therapy for IPF [46, 47]. In a controlled exposure of human subjects to DEP, oral NAC pre-treatment reduced airway responsiveness in hyperresponsive individuals [48]. However, as previously mentioned with vitamin C, NAC and vitamin C oral pre-treatment was found to augment DEP-induced vasoconstriction [49]. This indicates oral antioxidants may lead to unwanted systemic side effects in combination with air pollution exposure. No human studies were found using inhaled NAC to protect against air pollution; however, several in vitro cell culture and in vivo animal model studies have investigated the direct treatment of airway cells with NAC.

A recent study from Oh et al. explored using NAC-loaded microparticles to adsorb and remove particulate matter containing nitrates. The microparticles were found to effectively adsorb nitrate and were able to be cleared following intratracheal instillation in mice. The results from this study are promising and warrant further testing using more functional markers of protection (e.g., BALF inflammatory cells, oxidative stress, lung function). Therapeutic effects have also been reported in the context of ozone and nitrogen dioxide exposure. Intravenous NAC pretreatment was found to prevent ozone-induced mucociliary dysfunction in sheep, and post-exposure intraperitoneal NAC treatment reversed airway hyperresponsiveness in mice [50, 51]. NAC was also able to abrogate cytokine release caused by combined rhinovirus infection and oxidant gas exposure [52].

Despite seemingly positive results in the previously mentioned studies, a review from 2007 on the induction of antioxidant enzymes to protect against the adverse effects of DEPs briefly mentioned the idea of using inhaled NAC to protect against inhaled oxidants; however, they reported that preliminary studies from their laboratory using inhaled NAC did not see any protective effects against DEPs in an in vivo human nasal model [53]. Given no other information or data was provided, further investigation into this concept is warranted. Another study using well-differentiated primary hBECs from a COPD cohort at an air–liquid interface (ALI) found that basolateral treatment with NAC following DEP exposure did not provide any significant therapeutic effects, but did show trends in reducing IL-8 secretion and antioxidant gene expression [54]. Other studies of DEP and PM, mostly in in vivo murine models and submerged cell cultures, reported anti-inflammatory and antioxidant effects of NAC pre- and post-exposure treatment [55–59]. Similar effects were also seen in response to benzo[a]pyrene-induced acute lung injury [60].

Interestingly, in an in vitro experiment comparing several antioxidants, including vitamin C, vitamin E, and NAC, all three reduced protein and lipid peroxidation, but NAC was the only one that improved the ratio of reduced to oxidized glutathione [61]. Considering NAC directly contributes to production of glutathione, this result is not surprising. A more unbiased measure of protective effects could provide more insightful data such as a copper-based total antioxidant capacity or cellular ROS assay. The same study also found that NAC protected ovalbumin-sensitized mice against DEP exposure, although vitamin C and E were not examined in this experiment.

### Melatonin

Melatonin is an endogenous hormone primarily synthesized in the pineal gland from tryptophan [62]. Melatonin biosynthesis is synchronized with the cycle of light and dark with serum melatonin concentrations following a circadian rhythm, playing a direct role in the body’s sleep cycle and thermoregulation [63]. Due to its antioxidant and anti-inflammatory properties, it has been investigated as a therapeutic for asthma, COPD, and allergic airway inflammation [63, 64].

Few human studies have been conducted on the effect of melatonin in the lungs. Cavalcante et al. conducted a randomized, double-blind, placebo-controlled study of oral melatonin in COPD patients which showed that melatonin administration reduced oxidative stress and relieved dyspnea (difficulty breathing) [65]. Notably, in a previous study using a similar dose and time of administration, melatonin improved sleep in moderate to severe COPD patients, with no detrimental effects on daytime alertness, lung function, and exercise capacity [66].

Although not as extensively studied as the previously mentioned antioxidants, melatonin has been shown to exhibit protective effects against PM and ozone. Intragastric melatonin alleviated PM_2.5_-induced lung injury, edema, ferroptosis, and lipid peroxidation through expression of Nrf2 [67]. Lee et al*.* found intraperitoneal melatonin protected against PM_2.5_ and acute ischemic reperfusion injury through reduction of oxidative stress, inflammation, and tracheal immune cell infiltration in mice [68]. Protective effects were also seen in PM_2.5_-exposed guinea pigs, along with a reduction of chronic cough following melatonin treatment [69]. In a murine model of ozone exposure, oral melatonin reduced oxidative stress and stabilized the Nrf2 pathway [70].

Melatonin has also shown promise in models of lung disease exacerbated by pollutant exposure. Two studies from Shin et al. have found that melatonin is able to attenuate MUC5AC secretion and gene expression in H292 cells and a murine model of asthma via intraperitoneal treatment [71, 72]. MUC5AC is one of the predominant mucins overexpressed in muco-obstructive lung diseases and in response to inhaled pollutants [73, 74]. The same group saw similar effects in vitro and in vivo in a cigarette smoke extract (CSE) and LPS model of COPD, along with suppression of pulmonary fibrosis [75]. This murine model was also used in a transcriptomic study of oral melatonin which found that melatonin alleviated lung damage and inflammation and reduced necroptosis [76]. Melatonin has also exhibited antiviral properties in various tissue. Huang et al. showed that oral melatonin inhibited lung oxidative stress, proinflammatory cytokine production, and inflammatory injury in RSV-infected mice [77].

### Vitamin C

Vitamin C, or ascorbic acid, is a water-soluble nutrient vital for proper immune function that cannot be synthesized endogenously. As one of the antioxidants found in ASL, many early studies investigating the effect of diet on susceptibility to air pollution included vitamin C. Several epidemiologic and exposure chamber studies that analyzed ozone pollution in healthy and asthmatic subjects found that oral supplementation of vitamin cocktails containing vitamin C, E, and/or β-carotene above the daily minimum requirement may provide protection against ozone-induced decreases in lung function and bronchoconstriction [78]. However, an in vivo exposure chamber study of diesel exhaust found that vitamin C and N-acetylcysteine (NAC) supplementation increased vasoconstriction caused by exposure, and another study of acute exposure to ambient PM and/or ozone found no effects on cardiovascular outcomes. Most importantly, it has been shown that dietary vitamin C supplementation does not significantly affect ASL concentrations of ascorbic acid [79, 80]. Together, these data do not provide strong evidence for the use of dietary vitamin C supplementation to protect against cardiovascular and pulmonary injury induced by air pollution.

Preliminary investigations of vitamin C treatment prior to air pollution exposure in vitro have shown more promising results. Studies from Lee et al. in house dust mite (HDM) stimulated H292 cells, a pulmonary mucoepidermoid cell line, and Jin et al. in PM2.5 stimulated 16HBE cells found that co-exposure of HDM/PM2.5 with ascorbic acid reduced ROS levels and inhibited inflammatory responses [81, 82]. Despite little evidence of effectiveness through oral supplementation, the few in vitro studies of direct vitamin C treatment onto airway cells show possible antioxidant and antiviral properties.

Although there are few studies looking at vitamin C to protect against air pollution in vitro, several other studies exhibit relevant effects without pollutant exposure. A study in human nasal epithelial cells (hNECs) exhibited an initial increase in cilia beat frequency following vitamin C treatment [83]. This suggests that intranasal administration of vitamin C may be capable of improving mucociliary clearance of inhaled pollutants. Transcriptional analysis of vitamin C-treated BEAS-2B cells, a bronchial epithelial cell line, found that pathways associated with antiviral activity were upregulated while pathways associated with lung injury, inflammation, oxidative stress were downregulated [84]. In the same model it was found that treatment increased responses to polyinosinic:polycytidylic acid (poly I:C), an antiviral ligand, and type I interferons, further supporting the antiviral properties of vitamin C in airway cells and potentially providing a way to combat air pollution-induced dysregulation of antiviral immune responses.

### Vitamin E

Vitamin E is a fat-soluble antioxidant used by humans primarily in the form of α-tocopherol as well as other tocopherols such as γ-tocopherol, and is exclusively obtained through diet [85]. In the context of the lungs, alpha-tocopherol has been most commonly studied as an inhibitor of allergic inflammation in allergies and asthma [86]. Dietary intervention studies using vitamin E almost all included vitamin C as mentioned previously, but in a controlled ozone exposure study of oral vitamin E alone, no significant changes in lung function were observed; however, vitamin E is one of the few antioxidants to have been investigated through in vivo aerosol delivery.

Gao et al. conducted a study pre-treating human subjects with an intranasally delivered cocktail of antioxidant oils (soy oil, coconut oil, orange oil, aloe vera oil, peppermint oil, and vitamin E oil) followed by acute ozone exposure [87]. Oil treatment was able to attenuate ozone-induced nasal inflammatory response and increase baseline levels of antioxidant gene *HO-1* in the nasal mucosa. In the same study, the antioxidant oil was applied to a lung epithelial cell line, inducing expression of several antioxidant genes, activating NRF2, and mitigating pro-inflammatory endotoxin signaling.

Another study in mice saw similar upregulation of *Nrf2* and *Ho-1*, as well as alleviation of ozone-induced lung injury and oxidative stress [88]. Vitamin E also exhibits protective effects against other pollutants such as Benzo[a]pyrene (BaP), acrolein, and aluminum nanopowder by preventing oxidative stress [89–91]. In addition to airway epithelial cells, vitamin E supplementation has been shown to prevent allergen-induced NRF2 suppression in asthmatic alveolar macrophages and reduced ozone-induced cell death in fibroblasts [92, 93]. Several murine studies have also found that dietary α-tocopherol can improve immune function, preventing lung injury and susceptibility to bacterial infection [94–96].

### Limitations of current research

The majority of clinical trials and in vivo murine experiments investigating antioxidant treatment to protect against air pollution-induced lung injury use either dietary supplementation or systemic delivery of antioxidants. Aerosol delivery presents several benefits for this purpose. Inhalation avoids hepatic first-pass metabolism and the lungs have lower enzymatic activity than other organs [97]. This prevents premature drug degradation and allows for lower doses to be used for direct delivery to the lungs. Direct delivery and absorption also aid in preventing unwanted system effects. Inhaled delivery presents a more convenient and less invasive option than intravenous delivery while also allowing for more direct treatment than oral delivery.

Current literature is also limited to mostly submerged cell lines and murine experiments using non-inhalational routes o exposure. Submerged cell cultures lack many of the physiological features of the airways such as the over 40 different cell types and variations in epithelial thickness, among others [98]. Exposure also presents a challenge as normally air-borne particles and gases are added directly into the cell culture media allowing for agglomeration of particles, reaction with the media, and difficulty assessing dose. A more physiologically relevant model is the culturing of primary airway cells at an air–liquid interface using permeable membrane supports. Although immortalized cell lines can be cultured at an ALI, primary cells are able to differentiate, forming a pseudostratified epithelium. Several exposure systems now exist that allow for apical deposition of air pollution onto ALI cultures and should be used to validate past results from submerged systems. ALI cultures, combined with in vitro nebulizer systems would allow for the most relevant model of inhaled delivery and air pollutant exposure through apical delivery of both pollutants and antioxidant aerosols [99].

Similarly, with murine models, nose-only and whole-body inhalation chambers can be used to test antioxidant aerosol pre-treatment followed by air pollution exposure in a whole body gas or PM exposure chambers [100, 101]. Mouse models would also provide useful data on immune effects as well as other systemic effects of antioxidant inhalation, as evidenced by previous studies using vitamin D. However, mice have several key lung morphological and physiological differences compared to humans such as breathing pattern (mice are primarily nose-breathers), cell composition (mice largely lack submucosal glands except in the trachea), and eosinophil function and distribution [102]. The gold standard to evaluate the ability of antioxidant inhalation to protect against air pollution would be to conduct controlled studies in environmental exposure chambers with human subjects. Epidemiological cohort studies utilizing ambient air quality data and various antioxidant treatment groups could also provide useful data regarding chronic, low-dose exposures. The same methodology previously used for dietary antioxidant studies can be applied to inhaled antioxidants, although inter-subject differences in inhalation technique, breathing patterns, and variability in nebulizer output present unique challenges to aerosol treatment.

### Considerations for inhaled antioxidant therapies

Regarding inhaled therapies, it is important to acknowledge that agents may have different effects when they are inhaled than when administered through oral or dermal routes of exposure. For example, flavoring compounds and humectants used in vaping products, although generally recognized as safe for oral consumption, can cause adverse effects when inhaled, especially following heated aerosolization and subsequent degradation/oxidation. Most notably is the example of inhaled vitamin E acetate, a compound safe for oral and dermal routes of exposures, but linked to e-cigarette or vaping use-associated lung injury (EVALI) as the potential causative component [103]. There are also examples of oral medications that when being used experimentally via inhalation cause significant toxicity, such as the death of a research participant following inhalation of hexamethonium [104]. Additionally, given the important role of redox homeostasis and the various beneficial roles of ROS/RNS in cellular function (signaling, oxidative burst, phagocytosis, mitogenic responses), antioxidant therapies are not guaranteed to produce positive results in human studies and may even result in detrimental effects [105–108]. For example, the Carotene and Retinol Efficacy Trial (CARET), a study of beta-carotene and retinyl palmitate supplementation, was stopped prematurely due to an increase in lung cancer risk and death from lung cancer in participants who received the intervention [109]. As such, critically evaluating local and system effects of inhaled antioxidant therapies is imperative.

## Conclusions and future directions

Based on the current body of evidence, antioxidants have the potential to provide protection against air pollution. In addition to directly scavenging ROS, several of these antioxidants also exhibit anti-inflammatory and antimicrobial properties, indicating they could alleviate oxidative stress as well as susceptibility to viral and bacterial respiratory infections caused by exposure to air pollution. Despite mixed results from dietary antioxidant studies, a limited number of studies using intranasal and intrapulmonary delivery show that these routes may prove more effective. Additionally, inhaled delivery avoids first pass metabolism, provides direct treatment, and potentially limits systemic off-target effects making it ideal for pulmonary protection [97]. Future clinical work should be done to examine the effects of inhaled antioxidants on air pollution exposure in controlled environmental exposure chamber studies and cohort studies using ambient air conditions. Additional in vitro work should be done to elucidate mechanisms of these antioxidants, especially in well-differentiated primary airway cells at an ALI as many previous studies were in cell lines and undifferentiated primary cells. Primary airway cells at ALI can also use cells from specific donors, such as asthmatics, which would provide information on protective effects for susceptible populations. In summary, antioxidant inhalation has promise as a possible preventative intervention for people with lung diseases like COPD, asthma, and cystic fibrosis, in which ASL antioxidant composition is altered and therefore warrants further investigation.

## Data Availability

Not applicable.

## Abbreviations

ASL: Airway surface liquid
ALI: Air–liquid interface
COPD: Chronic obstructive pulmonary disease
WHO: World health Organization
PM: Particulate Matter
AED: Aerodynamic equivalent diameter
ROS: Reactive oxygen species
RNS: Reactive nitrogen species
NAC: N-acetylcysteine
hNEC: Human nasal epithelial cell
poly I:C: Polyinosinic:polycytidylic acid
HDM: House dust mite
BaP: Benzo[a]pyrene
CYP27B1: 1α-Hydroxylase
RSV: Respiratory syncytial virus
hBEC: Human bronchial epithelial cell
LPS: Lipopolysaccharide
BALF: Bronchoalveolar lavage fluid
TH17: T helper 17
DEP: Diesel exhaust particle
EMT: Epithelial-mesenchymal transition
CSE: Cigarette smoke extract
GSH/GSSG: Glutathione (reduced/oxidized)
CF: Cystic fibrosis
IPF: Idiopathic pulmonary fibrosis
